# The complete mitochondrial genome of *Hyporhamphus dussumieri* (Beloniformes; hemiramphidae) and phylogenetic studies of Beloniformes

**DOI:** 10.1080/23802359.2018.1532338

**Published:** 2018-10-26

**Authors:** Kehua Zhu, Zhenming Lü, Liqin Liu, Li Gong, Lihua Jiang, Bingjian Liu

**Affiliations:** a National Engineering Laboratory of Marine Germplasm Resources Exploration and Utilization, Zhejiang Ocean University, Zhoushan, China;; b National Engineering Research Center for Facilitated Marine Aquaculture, Marine science and technology college, Zhejiang Ocean University, Zhoushan, China

**Keywords:** *Hyporhamphus dussumieri*, mitogenome, phylogenetic tree

## Abstract

The *Hyporhamphus dussumieri* is a kind of minor commercial fishes, usually marketed fresh and dried salted. However, to date, there is a limited genetic resource available for this species. In this study, we assembled the whole mitochondrial genome of this species yielding a 16,542 bp circular assembly composed of the typical vertebrate mitochondrial features. It contains 13 protein-coding genes, two rRNA genes, 22 tRNA genes, a putative control region, and one origin of replication on the light-strand. The overall base composition includes C(28.3%), A(28.9%), T(27.1%), and G(15.7%). Moreover, the 13 PCGs encode 3800 amino acids in total. The result of the phylogenetic tree supports *H. dussumieri* has a closest relationship with *Hemiramphus far*.

The Dussumier’s halfbeak (*Hyporhamphus dussumieri*) lives in reefs and shallow lagoons. They live solitary or in groups, and are often seen circling snorkelers, just under the surface of the water (Fricke et al. 2011), most common around islands and marketed fresh and dried salted (Carpenter and Carpenter 2002). Genetic resources for this species are presently lacking. In this study, we described the complete mitochondrial genome of *H. dussumieri* and explored the phylogenetic relationship within Beloniformes, to gain its molecular information and thus contribute to facilitate future studies on population genetic structure and phylogenetic relationships.

Here, we assembled the mitogenome of *H. dussumieri* by Codoncode Aligner, which was sampled from Tsankiang, China (21°16'25"N; 110°21'16"E) and stored in laboratory of Zhejiang Ocean University with accession number 20150826dsxz22. Similar to the typical mitogenome of vertebrates (Zhu et al. 2018a, 2018c), the mitogenome of *H. dussumieri* is a closed double-stranded circular molecule of 16,542 nucleotides (GenBank accession no. MH734933), within the range of other teleost mitogenomes. The complete mitochondrial genome contains 13 protein-coding genes (PCGs), two ribosomal RNA genes (12S rRNA and 16S rRNA), 22 transfer RNAs (tRNA) genes, and a putative control region (CR) and one origin of replication on the light-strand (OL). The overall base composition is 28.9% A, 28.3% C, 27.1% T, and 15.7% G, respectively, with a slight AT bias (56.0%). Most mitochondrial genes of *H. dussumieri* were encoded on the H-strand, with only ND6 and eight tRNA (Gln, Ala, Asn, Cys, Tyr, Ser-UCN, Glu, and Pro) genes encoded on the L-strand. 13PCGs genes encode 3800 amino acids in total, all of them use the initiation codon ATG except COI starts with GTG, which is quite common in vertebrate mtDNA (Miya et al. 2001; Zhu et al. 2018b). Most of them have TAA or TAG as the stop codon, whereas three protein-coding genes (COII, ND4, Cytb) ended with a single T. The lengths of 12S rRNA located between tRNA^Phe^ and tRNA^Val^ and 16S rRNA located between tRNA^Val^ and tRNA^Leu^ were 946 bp and 1691 bp, respectively. The origin of light-strand replication is located in a cluster of five tRNA genes (WANCY), which has the potential to fold into a stable stem-loop secondary structure, with a stem formed by 13 paired nucleotides and a loop of 13 nucleotides; the CR is determined to be 876 bp, which is located between the tRNA-Pro and tRNA-Phe genes, and three typical domains are observed, including the TAS, central CSB and CSB, which is identical to that in other teleostean mitogenomes (Zhang et al. 2013).

The phylogenetic tree based on the neighbour-joining method was constructed to provide relationship within Beloniformes. The result of the present study supports *H. dussumieri* has a closest relationship with *Hemiramphus far*, highly supported by a bootstrap probability of 92% (Figure 1), this characteristic likely contributes to the high population genetic structuring typically observed in species belonging to Hemiramphidae (Meisner and Burns 1997; Tibbetts et al. 2007).

**Figure 1.**
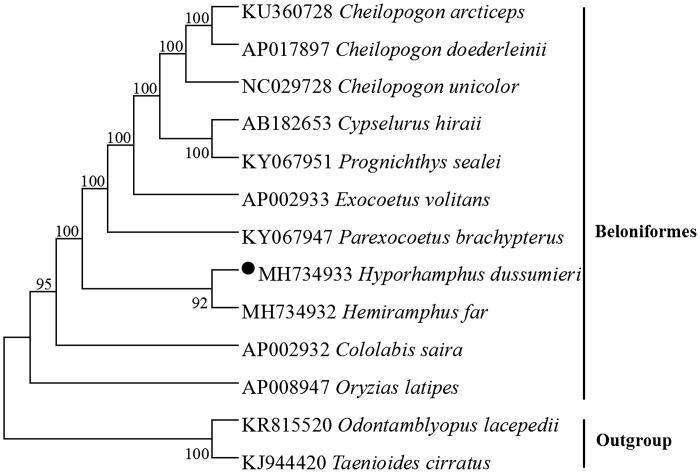
Neighbour-joining (NJ) tree of 11 Beloniformes species based on 12 PCGs encoded by the heavy strand. The bootstrap values are based on 1000 resamplings. The number at each node is the bootstrap probability. The number before the species name is the GenBank accession number. The genome sequence in this study is labeled with a black spot.
